# Glycemic Disturbances in Pheochromocytoma and Paraganglioma

**DOI:** 10.7759/cureus.4551

**Published:** 2019-04-27

**Authors:** Joshua A Ronen, Meredith Gavin, Misty D Ruppert, Alan N Peiris

**Affiliations:** 1 Internal Medicine, Texas Tech University Health Sciences Center, Odessa, USA; 2 Internal Medicine, Texas Tech University Health Sciences Center, Lubbock, USA

**Keywords:** pheochromocytoma, paraganglioma, extra-adrenal pheochromocytoma, hyperglycemia, hypoglycemia, neurofibromatosis type 1, von hippel lindau, multiple endocrine neoplasia ii, insulin sensitivity, insulin resistance

## Abstract

In this review article, we aimed to analyze the available data on pheochromocytomas and paragangliomas as it pertains to their not as well-recognized association with significant glycemic abnormalities in the preoperative, perioperative, and postoperative settings as well as how they should be managed clinically. Pheochromocytomas are rare adrenal tumors that account for about 0.1% of hypertension. Paragangliomas, on the other hand, are even less common and have fewer clinical manifestations. Both types of tumors may have unusual modes of presentation which can challenge even the most experienced clinicians and are easy to overlook, resulting in post-mortem diagnosis. We wish to draw further attention to the life-threatening effects on glucose and insulin homeostasis that can occur in the form of hyperglycemic and hypoglycemic states. Hyperglycemia is a result of a glucose intolerant state created in the setting of catecholamine excess, which can present in the form of resistant diabetes, diabetic ketoacidosis (DKA), or even hyperglycemic hyperosmolar states (HHS). In many reported cases, these abnormalities resolve with resection of the tumor. However, past clinicians have also described a state of "reactive hypoglycemia" that can occur following tumor resection, further emphasizing the need for very close perioperative and postoperative monitoring. Severe hypoglycemia may also occur with inherited diseases linked to pheochromocytoma such as von Hippel-Lindau (VHL) disease as well as predominantly epinephrine-producing tumors, given some of the dramatic downstream effects of alpha and beta adrenoceptor agonization. While much of the data remains anecdotal, clinicians will benefit from the awareness of the protean manifestations of these tumors and the varied and lesser-known effects on glucose and insulin homeostasis.

## Introduction and background

Pheochromocytomas account for about 0.1% of hypertension and approximately 76% may be diagnosed post-mortem [1]. While many manifestations of pheochromocytoma in the setting of catecholamine excess are well described, the varied manifestations of disturbed glucose and insulin metabolism are not as well-recognized. Increased serum metanephrines in an asymptomatic pheochromocytoma may impact glucose and insulin parameters without raising blood pressure, as suggested by Uehara [2].

The catecholamines such as epinephrine and norepinephrine play an important part in countering hypoglycemia especially if glucagon release is impaired [3]. However, excess catecholamines can have deleterious effects on glucose and insulin homeostasis. Catecholamines promote increased insulin resistance, compromising insulin secretion and decreasing glucose uptake. This can present as glucose intolerance (a pre-diabetic state with increased risk of cardiovascular pathology) or one of frank diabetes mellitus (a fasting hyperglycemic state with a strong tendency toward ketosis). The predominant mechanism may be impaired insulin secretion, especially the early phasic release of insulin [4]. Catecholamines may also exert significant antagonistic effects on glucose utilization through beta-adrenoreceptor desensitization, increased lipolysis, and a pro-inflammatory state which can induce hepatic insulin resistance [5-7]. However, both reactive and fasting hypoglycemia may also occur in pheochromocytoma. In this clinical review, we discuss both facets of hyperglycemia and hypoglycemia. Each can pose serious clinical management issues if not addressed in a timely manner. Paragangliomas are tumors of neural crest tissue and are less likely to be symptomatic. These tumors can also be associated with catecholamine excess and are discussed. Functional hormonal disturbances including diabetes can occur but are less frequent than in pheochromocytoma [8]. 

## Review

Variable hyperglycemic presentations

The association of diabetes with pheochromocytoma is not new [9]. Diabetes can be the presenting feature of pheochromocytoma irrespective of tumor location [10]. Larger symptomatic pheochromocytomas may present as diabetes in 23% to 33% of patients [11-12]. Glucose intolerance may be present in about 50% of pheochromocytoma patients [13]. Epinephrine-secreting pheochromocytomas may be more likely to induce glucose intolerance or frank diabetes mellitus by the preferential affinity for the β-receptor. Beta-receptors stimulate gluconeogenesis and α-2 receptors decrease insulin release. These may be significant contributors to pheochromocytoma-associated hyperglycemia [14]. Catecholamines may induce insulin resistance through desensitization of beta-adrenergic receptor [5]. Moving forward in this discussion, it is important to consider the physiologic effects of norepinephrine (α-1 and α-2) and epinephrine (α-2, β-1, and β-2) and how they influence the pathology described, given the respective locations of the adrenoreceptors themselves.

Endogenous catecholamine excess in patients with pheochromocytoma can induce or aggravate insulin resistance in patients with either normal glucose tolerance or type 2 diabetes [15]. The presence of diabetes in young, lean hypertensive patients may be a clue to the underlying diagnosis [16]. Resistance to standard diabetes therapy may also be a feature of pheochromocytoma, even when hypertension is controllable [17-18]. Pheochromocytoma may be rarely associated with insulin-dependent diabetes that resolves post-resection [19]. Still, diabetic ketoacidosis (DKA) has been reported in pheochromocytoma such that the presence of marked hypertension at presentation of DKA may be a clue to an underlying pheochromocytoma [20-21]. Presentations such as hyperglycemic hyperosmolar states (HHS) have also been reported [22].

Vance et al. confirmed glucose intolerance with pheochromocytoma as a result of inhibition of insulin release. They also concluded that α-blockade may reverse both impaired glucose tolerance and insulin release [23]. In contrast, Diamanti-Kandarakis et al. reported only mild improvement in glucose utilization with α and β adrenergic receptor blocking agents [24]. They found that these agents appeared more effective in addressing the cardiovascular manifestations of pheochromocytoma. Colwell indicated that tumor removal was more effective than phentolamine, an alpha-blocker, for restoring normal glucose and insulin homeostasis and suggested that effects on insulin secretion may not be mediated entirely through α-adrenergic receptor stimulation [25]. Resection usually leads to resolution of diabetes unless other predisposing factors are present [11].

The possibility of multiple ectopic hormone production such as somatostatin and ACTH (or more rarely corticotrophin releasing hormone) from a pheochromocytoma may be an additional mechanism contributing to glucose intolerance [26].

Fasting and reactive hypoglycemia

Hypoglycemia is a frequent complication of pheochromocytoma resection. In 1977, Allen reported severe hypoglycemia following removal of pheochromocytoma in a single patient. He speculated that increased insulin release may have contributed to this phenomenon [27]. Wilkins confirmed occurrence of hypoglycemia in the immediate post-operative period following resection of a pheochromocytoma [28]. Hypoglycemia may occur with no discernible difference between adrenal and extra-adrenal pheochromocytomas regarding its propensity [29].

Sagalowsky indicated that preoperative α-blockade without concomitant β**-**blockade may predispose to hypoglycemia following tumor removal [30]. Studies using adrenergic stimulation in patients with pheochromocytoma indicated that hypoglycemia occurred less since proinsulin, the secretory and less active form of insulin, predominates [31]. Bolli et al. reported hypertensive crises during postprandial hypoglycemia in a patient with pheochromocytoma and concluded that hypoglycemia mediated a marked release of catecholamines. They concluded that glucagon secretion in response to hypoglycemia may be impaired by chronically high levels of catecholamines [32].

Costello reported hypoglycemia after resection of pheochromocytoma and implicated enhanced insulin release due to the sudden withdrawal of catecholamines and beta-blocker use. This resulted in reduced glucagon secretion and hepatic glucose production [33]. Sagalowsky implicated phentolamine, a non-selective α-blocker, as a potential contributor to hypoglycemia. However, evidence of hypoglycemia with α-blockade is not well documented [30].

Akiba reported severe hypoglycemia in 13% of pheochromocytoma patients, usually two to 4.5 hours after surgery [34]. Patients with higher preoperative urinary epinephrine, but not norepinephrine, and those with either diabetes mellitus or impaired glucose tolerance were more likely to develop postoperative hypoglycemia [29]. Pheochromocytoma and paragangliomas may be linked to multiple endocrine neoplasia (MEN) type 2, von Hippel-Lindau (VHL) disease, and neurofibromatosis type 1 (NF1). Severe hypoglycemia appears more prevalent in VHL disease. High tumor burden and increased hemodynamic volatility are associated with the greatest risk of fatal hypoglycemia [35-36]. Improved beta cell function and a comparison of pre-surgery to post-surgery status indicated a significant positive association to improved urinary metanephrines and a significant negative association to urinary normetanephrines [37]. Chen also reported that hypoglycemia may be more common in epinephrine-secreting tumors and after longer durations of operation [38]. Rarely, progressive and fatal hypoglycemia due to direct tumor consumption of glucose, as evidenced by positron emission tomography (PET), has been reported in malignant pheochromocytoma [39]. 

All patients undergoing resection of adrenal and extra‐adrenal pheochromocytomas require intensive monitoring of serum glucose levels during and after surgery. Intravenous glucose infusion with glucose monitoring is recommended. While there has been an emphasis on hypoglycemia following resection of pheochromocytomas, post-prandial hypoglycemia has also been noted. Hiramatsu reported reactive hypoglycemia and hyperinsulinemia following oral glucose in a patient with pheochromocytoma [40]. Stimulation of insulin production through activation of beta-2 adrenoceptors may also impact glucagon secretion and contribute to reactive hypoglycemia [41].

Paraganglioma is a rare tumor of the neural crest and can also be associated with catecholamine excess. It can have malignant potential and serial imaging is recommended for observation upon tissue diagnosis. Hypoglycemia has also been reported in paragangliomas, and co-secretion of insulin and catecholamines may rarely occur [42-43]. Interestingly, insulin growth factor II (IGF-II) has been identified in paraganglioma and pheochromocytoma tissue [44]. While IGF-II has been implicated in non-insulin mediated hypoglycemia in many tumors, no reports to date indicate this mechanism operates in pheochromocytomas and paragangliomas.

## Conclusions

Clinicians may be faced with life-threatening disturbances in glucose and insulin homeostasis when managing pheochromocytomas and/or paragangliomas. Hyperglycemic syndromes as well as reactive and fasting hypoglycemia as described may be encountered. Much of the data relating to pheochromocytoma, paraganglioma, and altered glucose and insulin homeostasis remain anecdotal. We recommend multicenter international collaborative studies be undertaken to better delineate the pathophysiology. Such studies would also be able to separate intrinsic catecholamine effects from medication and other factors that could also contribute to the altered hormonal milieu. Moreover, pheochromocytoma linked to neurocutaneous disorders such as neurofibromatosis and von Hippel-Lindau disease may have special characteristics that await delineation.
